# Case report: Spontaneous remission of severe aplastic anemia mediated by mutant hematopoietic stem cells evading T-cell attack

**DOI:** 10.3389/fimmu.2025.1635943

**Published:** 2025-10-01

**Authors:** Kohei Shiroshita, Yoshitaka Zaimoku, Himari Kudo, Naoshi Obara, Kazuyoshi Hosomichi, Yui Kano, Miku Kobayashi, Eriko Morishita, Hiroyuki Takamatsu, Takaaki Toyama, Shinji Nakao

**Affiliations:** ^1^ Department of Hematology, Federation of National Public Service Personnel Mutual Aid Associations Tachikawa Hospital, Tokyo, Japan; ^2^ Department of Hematology, Kanazawa University, Kanazawa, Japan; ^3^ Innovative Clinical Research Center, Kanazawa University Hospital, Kanazawa, Japan; ^4^ Department of Medical Science, Faculty of Medicine, University of Tsukuba, Tsukuba, Ibaraki, Japan; ^5^ Laboratory of Computational Genomics, School of Life Science, Tokyo University of Pharmacy and Life Sciences, Tokyo, Japan; ^6^ Department of Clinical Laboratory Science, School of Health Sciences, College of Medical, Pharmaceutical and Health Sciences, Kanazawa University, Kanazawa, Japan; ^7^ Department of Clinical Laboratory Science, Division of Health Sciences, Graduate School of Medical Science, Kanazawa University, Kanazawa, Japan; ^8^ Faculty of Transdisciplinary Sciences for Innovation, Institute of Transdisciplinary Sciences for Innovation, Kanazawa University, Kanazawa, Japan

**Keywords:** severe aplastic anemia, spontaneous remission, immune escape, HLA loss, paroxysmal nocturnal hemoglobinuria, somatic mutation, chromosome 6p loss of heterozygosity

## Abstract

T-cell-mediated severe aplastic anemia (SAA) is typically fatal without prompt hematopoietic stem cell transplantation or intensive immunosuppressive therapy. Although rare cases of spontaneous remission have been reported, the underlying mechanisms remain poorly understood. A 24-year-old woman was incidentally found to have mild pancytopenia during a routine workplace health checkup. Over the subsequent 12 months, her pancytopenia gradually worsened, resulting in exertional dyspnea, purpura, and a diagnosis of SAA. Remarkably, her blood counts began to improve spontaneously 11 days after the diagnosis without any treatment or transfusions. She no longer met the criteria for SAA by day 27 and achieved complete hematologic normalization within three months. At 22 months, flow cytometry and targeted sequencing revealed that 69% of her granulocytes lacked the HLA-A*02:01-C*03:04-B*40:02-DRB1*14:54 haplotype due to acquired loss of heterozygosity, while 23% were glycosylphosphatidylinositol-deficient owing to *PIGA* mutations. Retrospective digital polymerase chain reaction of diagnostic bone marrow demonstrated that nearly all non-lymphoid cells had already been replaced by HLA allele-lacking clones, whereas glycosylphosphatidylinositol-deficient erythrocytes constituted only 0.25%. These findings suggest that hematologic recovery occurred through the selective expansion of mutant hematopoietic stem cells capable of evading persistent T-cell-mediated destruction. Early identification of HLA allele-lacking leukocytes may help predict spontaneous remission and avoid unnecessary intensive therapy in patients with SAA.

## Introduction

Aplastic anemia (AA) is a life-threatening bone marrow failure disorder characterized by pancytopenia resulting from a marked reduction in hematopoietic stem cells (HSCs) (1). Idiopathic severe AA (SAA) is most commonly caused by T cell-mediated destruction, for which prompt treatment with intensive immunosuppressive therapy (IST) or hematopoietic stem cell transplantation is essential for survival (2–4).

Although rare, spontaneous remission of SAA has been reported (5–7). Lee et al. described 18 cases of AA, including eight with SAA, in which spontaneous remission occurred at a median of 14 days (range, 4–332) (5). However, they concluded that most cases likely represented recovery from transient bone marrow suppression triggered by external factors, such as medications or infections, rather than true idiopathic SAA with an immune pathogenesis. Similarly, spontaneous remission of pregnancy-associated AA has been observed following delivery (6).

The peripheral blood of patients with immune-mediated SAA often contains progeny of mutant HSCs that evade T-cell attacks. These include blood cells deficient in glycosylphosphatidylinositol (GPI) due to *PIGA* mutations (8, 9), as well as cells lacking expression of HLA class I alleles due to copy-neutral loss of heterozygosity on chromosome 6p (6pLOH) or loss-of-function mutations in HLA genes (10–17). These immune escape HSC clones may contribute to spontaneous remission, although definitive evidence supporting this hypothesis is lacking.

We herein report the first documented case of spontaneous remission in idiopathic SAA, attributed to the concurrent expansion of HLA allele-lacking and GPI-deficient HSCs.

## Case description

A 24-year-old woman underwent a routine physical examination at her workplace every six months. Eighteen months prior to presentation, her blood count was within normal range. Twelve months prior, mild pancytopenia with macrocytic anemia was first detected: white blood cell count (WBC), 3.54 × 10^9^/L; hemoglobin, 92 g/L; mean corpuscular volume, 110 fL; and platelet count, 112 × 10^9^/L. Over the subsequent six months, her pancytopenia worsened: WBC, 2.71 × 10^9^/L; hemoglobin, 72 g/L; and platelet count, 59 × 10^9^/L. Despite developing exertional dyspnea and petechiae in her lower extremities, she did not seek medical attention, as her symptoms remained mild. Two weeks before her admission to our hospital, she visited a local clinic because of a fever that had lasted for a week. A blood test revealed worsening leukopenia (WBC, 2.10 × 10^9^/L) and thrombocytopenia (platelet count, 36 × 10^9^/L), prompting referral to our hospital for further evaluation.

By the time of admission, the fever had resolved. The patient had no history of medication use, chemical exposure, or menstrual irregularities. Physical examination was unremarkable, with no signs of bleeding tendency, hepatosplenomegaly, or congenital anomalies suggestive of inherited bone marrow failure syndrome. Laboratory tests revealed progression of pancytopenia with an inadequate reticulocyte response: neutrophil count, 0.53 × 10^9^/L; hemoglobin, 76 g/L; reticulocyte count, 36 × 10^9^/L; and platelet count, 15 × 10^9^/L (**Table 1**).

**Table 1:** Laboratory test results at the diagnosis.

Item	Value	Reference range
Hematology		
White blood cell count, ×10^9^/L	3.0	3.3–8.6
Neutrophil, %	17.8	40–70
Lymphocyte, %	76.3	20–50
Monocyte, %	5.3	2–9
Eosinophil, %	0.3	1–6
Basophil, %	0.3	0–2
Red blood cell count, ×10¹²/L	2.24	3.86–4.92
Hemoglobin, g/L	76	116–148
Mean corpuscular volume, fL	108	83.6–98.2
Mean corpuscular hemoglobin concentration, g/L	314	317–353
Reticulocyte, %	1.6	0.5–2.0
Platelet count, ×10^9^/L	15	158–348
Coagulation		
Fibrinogen, g/L	24.2	16–35
Prothrombin time–international normalized ratio	0.99	0.8–1.2
Activated partial thromboplastin time, sec	34.5	24–40
Biochemistry		
Total protein, g/L	72	66–81
Albumin, g/L	39	41–51
Total bilirubin, μmol/L	8.6	7–26
Aspartate aminotransferase, U/L	36	13–30
Alanine aminotransferase, U/L	63	7–23
Alkaline phosphatase, U/L	69	38–113
γ-Glutamyl transpeptidase, U/L	33	9–32
Lactate dehydrogenase, U/L	299	124–222
Blood urea nitrogen, mmol/L	3.93	2.86–7.14
Creatinine, μmol/L	48.6	40.7–69.8
Uric acid, μmol/L	161	155 – 327
Sodium, mmol/L	137	138–145
Potassium, mmol/L	3.6	3.6–4.8
Chloride, mmol/L	107	101–108
Calcium, mmol/L	2.05	2.20–2.53
Phosphate, mmol/L	2.6	2.7–4.6
Iron, μmol/L	38.8	7.2–33.7
Total iron binding capacity, μmol/L	53.7	44.0–73.4
Ferritin, μg/L	284.4	4.1–120.2
Vitamin B_12_, pmol/L	184.5	180–914
Folic acid, nmol/L	19.5	>4
Copper, μmol/L	25.2	10.7–20.1
Zinc, μmol/L	14.2	9.9–16.8
Inflammation and hormones		
C-reactive protein, mg/L	<1.4	<1.4
Immunoglobulin G, g/L	14.41	8.61–17.5
Immunoglobulin A, g/L	3.52	0.93–3.93
Immunoglobulin M, g/L	2.05	0.50–2.69
Thyroid-stimulating hormone, mIU/L	1.64	0.61–4.23
Free thyroxine, pmol/L	12.1	9.1–19.6
Erythropoietin, IU/L	1810	4.2–23.7

**Figure 1:**
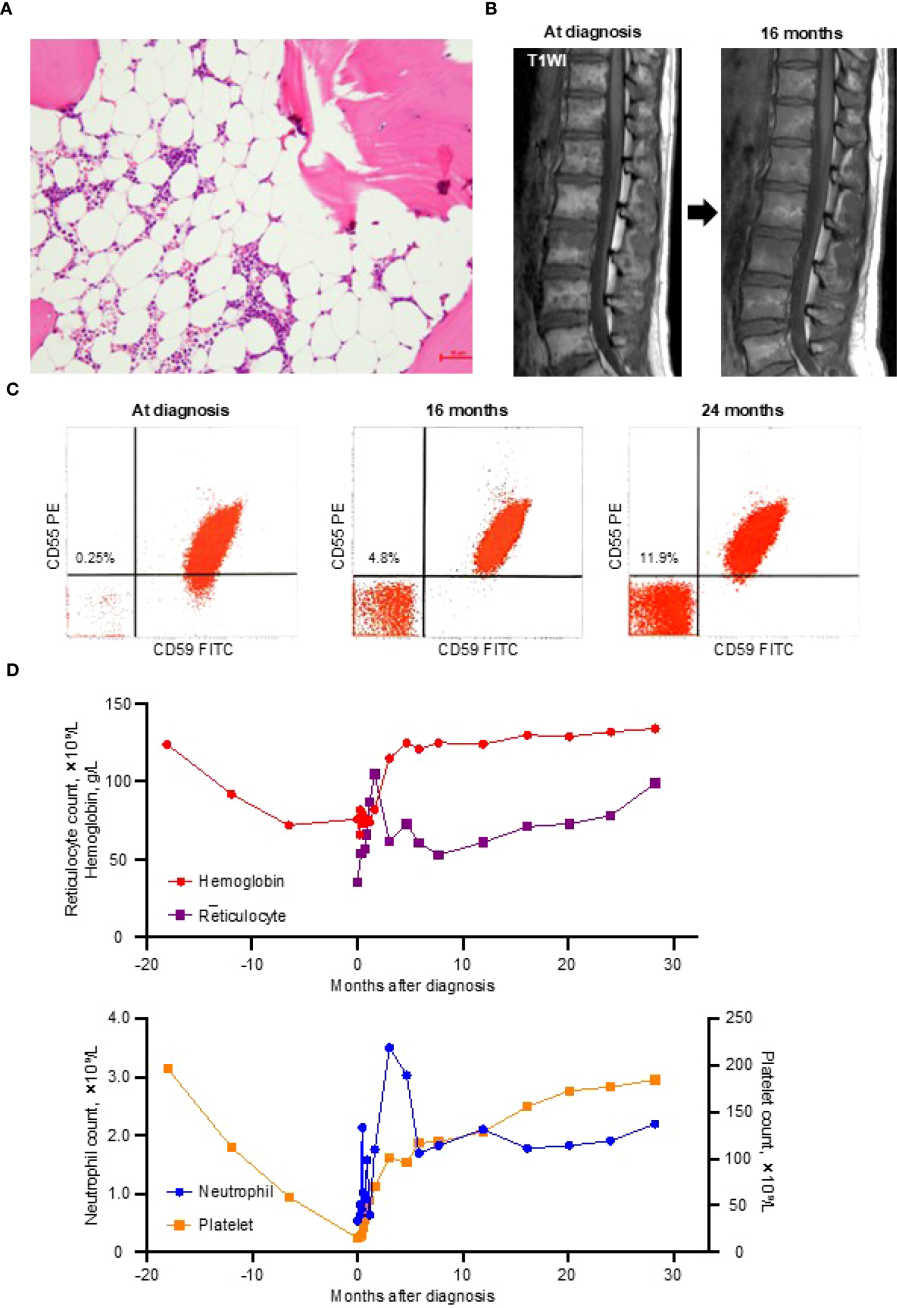
Diagnostic findings and the clinical course. **(A)** A bone marrow trephine biopsy at diagnosis showing severe hypocellularity with fatty replacement. **(B)** T1-weighted magnetic resonance imaging (T1WI) of the thoracolumbar spine demonstrating fatty marrow conversion (high signal intensity) at the diagnosis and improvement at 16 months. **(C)** Glycosylphosphatidylinositol-deficient (CD55^–^CD59^–^) erythrocytes detected by flow cytometry at the diagnosis and at 16 and 24 months thereafter. **(D)** Trends in blood cell counts from 18 months before the diagnosis to 28 months after the diagnosis. FITC, fluorescein isothiocyanate; PE, phycoerythrin.

Bone marrow examination revealed severe hypocellularity (**Figure 1A**), with no evidence of dysplasia or increased blasts, and cytogenetic analysis showed normal karyotype. Magnetic resonance imaging of the thoracolumbar spine revealed that most of the marrow space was replaced by fatty tissue, with patchy areas of residual hematopoietic activity (**Figure 1B**). Flow cytometry using anti-CD55 and anti-CD59 monoclonal antibodies identified 0.25% of erythrocytes and 0.2% of granulocytes as GPI-deficient (**Figure 1C**). These findings were all consistent with a diagnosis of idiopathic SAA.

During the 11-day diagnostic period, her reticulocyte and platelet counts increased to 54 × 10^9^/L and 18 × 10^9^/L, respectively, without any medication or blood transfusion. This unexpected improvement led the attending physician to withhold the planned IST. By day 27 after presentation, her blood counts no longer met the criteria for SAA (neutrophil count, 1.58 × 10^9^/L; hemoglobin, 76 g/L; reticulocyte count, 66 × 10^9^/L; platelet count, 46 × 10^9^/L), and all parameters normalized within three months (**Figure 1D**).

This hematologic recovery was accompanied by a gradual increase in GPI-deficient erythrocytes, reaching 4.8% at 16 months and 11.9% at 24 months (**Figure 1C**), without signs of intravascular hemolysis (lactate dehydrogenase at 24 months, 199 U/L). Follow-up magnetic resonance imaging at 16 months showed partial resolution of the fatty marrow changes (**Figure 1B**). As of May 2025, the patient has remained in complete remission without any treatment for 30 months.

## Diagnostic assessment

**Figure 2:**
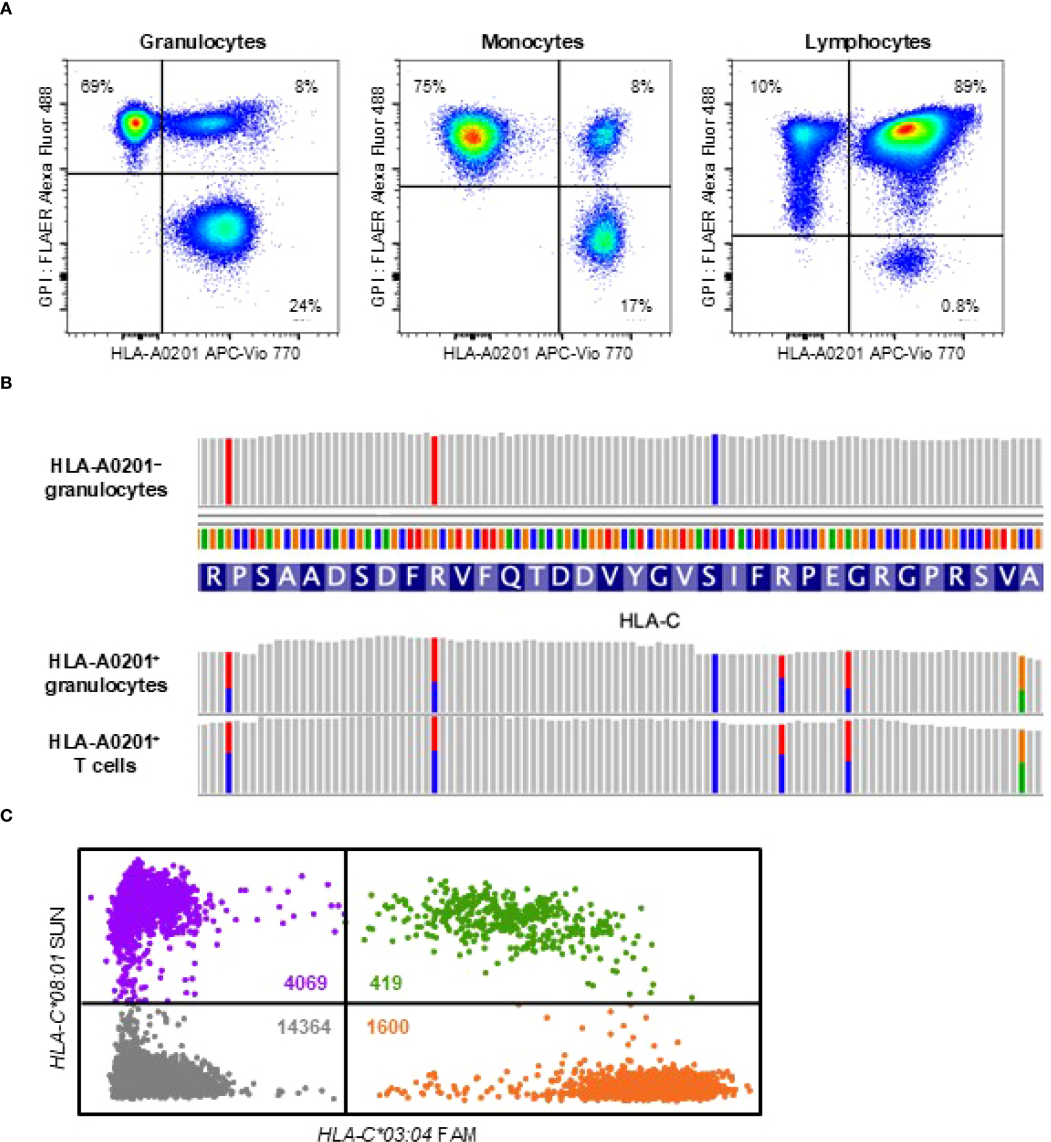
HLA loss analysis. **(A)** Flow cytometry at 22 months after the diagnosis showing HLA-A0201-lacking cells (left upper quadrant), GPI-deficient cells (right lower quadrant), and wild-type cells expressing both HLA and GPI (right upper quadrant) across three leukocyte subsets: high side-scatter (SSC^hi^) CD45^dim^CD33^dim^ granulocytes, high forward-scatter (FSC^hi^) CD45^hi^CD33^hi^ monocytes, and CD45^hi^CD33^–^ lymphocytes. **(B)** Targeted sequencing of HLA genes revealing loss of heterozygosity at the *HLA-C* locus in sorted HLA-A0201-lacking granulocytes, while heterozygosity was preserved in HLA-A0201-expressing granulocytes and T cells. **(C)** Digital polymerase chain reaction analysis of a bone marrow smear obtained at the diagnosis showing significantly fewer microwells containing HLA-C*03:04 (1600 orange dots) compared to those containing HLA-C*08:01 (4069 purple dots), indicating loss of HLA-C*03:04 due to 6pLOH. Green dots represent microwells containing both alleles and grey dots represent those with neither allele. The estimated concentrations of HLA-C*03:04 and HLA-C*08:01 in the reaction mixture, calculated using the Poisson distribution, were 240.6 copies/µL and 573.5 copies/µL, respectively. The clonal burden of the 6pLOH cells among total bone marrow nucleated cells was calculated to be 40.9% using the following formula: Clonal burden (%) = (573.5 − 240.6)/(573.5 + 240.6) × 100. APC, allophycocyanin; FAM, 6-carboxyfluorescein; FLAER, fluorescently labeled inactivated aerolysin; GPI, glycosylphosphatidylinositol.

To investigate the mechanism underlying her spontaneous remission, we assessed the presence of HLA allele-lacking and GPI-deficient cells 22 months after the diagnosis. Flow cytometry using anti-HLA-A2 monoclonal antibodies and fluorescently labeled inactivated aerolysin, as previously described (15), revealed that 69% of granulocytes and 75% of monocytes lacked HLA-A0201 expression. In addition, GPI-deficient cells retaining HLA expression accounted for 24% of granulocytes and 17% of monocytes (**Figure 2A**). In contrast, 89% of lymphocytes retained normal expression of both HLA and GPI, with only 10% lacking HLA-A0201 and 0.8% being GPI-deficient.

Targeted sequencing and HLA genotyping of sorted cell populations, using T cells expressing both GPI and HLA-A0201 as the germline control, revealed that all HLA-A0201-lacking granulocytes lost the HLA haplotype A*02:01-C*03:04-B*40:02-DRB1*14:54 due to 6pLOH (**Table 2**; **Figure 2B**). GPI-deficient granulocytes harbored two distinct *PIGA* frameshift mutations: c.577_581delGTACT (variant allele frequency, 43%) and c.845delA (variant allele frequency, 4%). No mutations in any of the 51 genes associated with myeloid malignancies were detected in the wild-type, GPI-deficient, or HLA-lacking granulocytes. At 28 months after the diagnosis—six months following the initial HLA loss analysis —the percentages of HLA-A0201-lacking and GPI-deficient cells remained unchanged.

**Table 2:** HLA genotyping results of sorted cell populations.

HLA locus	HLA-A0201^+^ T cells		HLA-A0201^–^ granulocytes	
	Allele 1	Allele 2	Allele 1	Allele 2
*HLA-A*	02:01:01	26:01:01	26:01:01	–
*HLA-B*	40:02:01	40:06:01	40:06:01	–
*HLA-C*	03:04:01	08:01:01	08:01:01	–
*HLA-DRB1*	14:54:01	09:01:02	09:01:02	–

Lost alleles in HLA-A0201-lacking granulocytes are indicated in bold.

To determine whether HLA allele-lacking cells due to 6pLOH were present at the time of diagnosis, we extracted genomic DNA from initial bone marrow smears and performed digital polymerase chain reaction to quantify allele-specific copy numbers of *HLA-C*, as previously described (12). This analysis revealed that 41% of bone marrow nucleated cells harbored 6pLOH (**Figure 2C**). Given that lymphocytes accounted for 62% of the nucleated bone marrow cells, it was estimated that nearly all non-lymphoid hematopoietic cells had already been replaced by HLA allele-lacking cells at the time of diagnosis. However, it is possible that bone marrow aspirates were obtained from a hematopoietic niche dominated by HLA-A0201-lacking stem cells, while wild-type cells continued to be produced at other sites, as cells expressing both HLA and GPI accounted for 8% of granulocytes and monocytes at 22 months after the diagnosis.

## Discussion

HSCs deficient in HLA class I or GPI can evade T-cell-mediated destruction and sustain clonal or oligoclonal hematopoiesis for an extended period following IST (12, 15, 18–21). However, all previously reported cases of remission with escape hematopoiesis had been treated with immunosuppressive therapy or anabolic steroids, spontaneous complete remission of SAA mediated by such immune-escaping HSC clones has not been previously reported. This phenomenon may be underrecognized, as most patients receive standard therapy shortly after diagnosis in accordance with current guidelines, and HLA loss is not routinely assessed in clinical practice.

HLA loss due to copy-neutral 6pLOH in AA was first identified in 2011 through copy number analysis using single nucleotide polymorphism array (10, 11). Although 6pLOH provides evidence of immune-mediated HSC depletion and can aid in diagnosing bone marrow failure of autoimmune origin (22–24), the relatively low prevalence of this abnormality (~13%) among AA patients limits its clinical utility (11, 25). More recent studies have identified loss-of-function mutations in HLA class I genes as an additional and more frequent mechanism of HLA loss (12–17). HLA allele-lacking leukocytes, arising from either 6pLOH or loss-of-function mutations, can be detected in 25-43% of patients with SAA using sensitive flow cytometry with HLA allele-specific monoclonal antibodies (15, 26). The impact of HLA loss on response to immunosuppressive therapy and prognosis of AA differs depending on the HLA class I alleles that is lost (13, 15).

In our case, T-cell-mediated HSC destruction appears to have begun 12–18 months prior to the diagnosis, potentially triggered by the loss of tolerance to self-antigens presented by HLA-A0201 or HLA-B4002 (11, 12). The gradual progression of the disease may have allowed sufficient time for the selective expansion of immune-evading HSC clones. The steep recovery observed during the first four weeks, which did not align with the subsequent hematologic recovery, may have been influenced by the fever the patient experienced prior to hospitalization. By the time of the diagnosis, most residual HSCs had been replaced by 6pLOH clones, along with a small population of GPI-deficient cells. These immune-escaping clones jointly restored hematopoiesis in the absence of any treatment, leading to complete recovery within three months. The absence of driver gene mutations in HLA-lacking or GPI-deficient granulocytes supports the notion that immune pressure alone was sufficient to drive their expansion.

In AA patients who harbor both HLA allele-lacking and GPI-deficient cell populations, one population can occasionally outcompete the other; however, it remains unpredictable which will preferentially expand (27). Nevertheless, we initially expected that GPI-deficient cells in the present case would eventually disappear during the expansion of HLA allele-lacking HSCs, based on a previously reported case of SAA treated with cyclosporine and methenolone (21). In that case, although the initial treatment response was poor, durable remission was ultimately achieved through the gradual expansion of HLA-A*02:06-deficient cells, accompanied by a decline and eventual disappearance of GPI-deficient cells. This pattern is thought to reflect the complete immune evasion of HLA-lacking HSCs from antigen-specific CD8^+^ T-cell-mediated cytotoxicity (22), whereas GPI-deficient HSCs, which retain HLA expression, remain partially susceptible to CD8^+^ T-cell attack.

In contrast, in our case, GPI-deficient cells gradually expanded during spontaneous recovery. This observation may indicate the coexistence of a distinct, yet incompletely understood, immune mechanism—such as CD4^+^ T cell-mediated marrow suppression—that selectively spares GPI-deficient HSCs while targeting HLA class I allele-lacking HSCs (28–30). This raises concerns about the potential for future relapse and progression to paroxysmal nocturnal hemoglobinuria and highlights the need to explore therapeutic strategies specifically targeting this mechanism.

This case report provides valuable clinical insight. A high percentage of HLA allele-lacking leukocytes at the time of the AA diagnosis may predict spontaneous remission. Even when the percentage is low, treatment with thrombopoietin receptor agonist, with or without cyclosporine, might facilitate the proliferation of immune-escaping HSCs and reduce the need for intensive IST or hematopoietic stem cell transplantation. Therefore, detection of HLA-lacking leukocytes may be considered as part of the diagnostic evaluation of AA to help assess the potential for spontaneous remission. Further studies are needed to clarify the incidence and long-term outcomes of spontaneous remission mediated by the immune-evading mutant HSCs. A nationwide prospective study is currently underway to validate the prognostic value of detecting the immune-escaping clones in treatment-naïve patients with AA.

## Data Availability

All data supporting the findings of this study are included in the article and its supplementary information files. Additional details are available from the corresponding author upon reasonable request.
